# Breast Cancer Epidemiology among Lebanese Women: An 11-Year Analysis

**DOI:** 10.3390/medicina55080463

**Published:** 2019-08-10

**Authors:** Mohamad Y. Fares, Hamza A. Salhab, Hussein H. Khachfe, Hassan M. Khachfe

**Affiliations:** 1 Faculty of Medicine, American University of Beirut, Beirut 1107, Lebanon; 2 Neuroscience Research Center, Faculty of Medical Sciences, Lebanese University, Beirut 1102, Lebanon; 3 School of Arts and Sciences, and the Lebanese Institute for Biomedical Research and Application (LIBRA), Lebanese International University (LIU), Beirut 1105, Lebanon

**Keywords:** breast cancer, cancer incidence, oncology, epidemiology, Lebanon

## Abstract

*Background and Objectives*: Breast cancer is the most prevalent cancer in women worldwide. Lebanon is a developing country in the Middle East with a prominent breast cancer incidence. The aim of our study was to explore the incidence rates of breast cancer in Lebanon from 2005 to 2015, and compare them to the rates of other countries. *Materials and Methods*: Breast cancer data for the years 2005–2015 was collected from the National Cancer Registry of Lebanon and stratified by gender and age group. Age-specific and age-standardized incidence rates were calculated and analyzed using joinpoint regression. Age-standardized incidence rates in the world population (ASR(w)) were obtained for other countries, from two online databases. *Results*: Breast cancer was found to be the most prevalent cancer in Lebanon, accounting for 20% of all cancer cases. The average ASR(w) was 96.5 per 100,000. Over the studied period, breast cancer ASR(w) in Lebanon showed a significantly increasing trend with an annual percent change (APC) of +4.6. Moreover, the APC of breast cancer age-specific rates significantly increased for the age groups 45–49 (*p* = 0.013), 50–54 (*p* < 0.001), 55–59 (*p* = 0.001), 60–64 (*p* = 0.002), 65–69 (*p* = 0.003), 70–74 (*p* < 0.001), and 75+ years (*p* < 0.001). Lebanon had the highest breast cancer ASR(w), when compared to other regional countries, and trailed only behind Denmark, when compared to selected countries from different parts of the world. *Conclusions*: Breast cancer incidence in Lebanon is among the highest in the world. Future studies should focus on exploring the genetic profile of the Lebanese population in an aim to extrapolate proper prevention guidelines.

## 1. Introduction

Breast cancer is the most prevalent cancer in women worldwide, and as a result, constitutes a major public health issue [1]. Annually, 1,384,155 new cases of breast cancer emerge and nearly 459,000 patients die due to this disease, making it one of the most fatal diseases globally [2,3,4]. While breast cancer incidence rates are higher in populations with high socioeconomic status, mortality and deaths are higher in populations with low socioeconomic status, and consequently, there exists a variation in breast cancer incidence and mortality between different regions and countries [5].

Breast cancer is considered highly heterogenous in etiology, pathology, and outcomes with some cases showing great prognosis and others showing aggressive clinical results [6,7,8,9,10]. Multiple reproductive and health risk factors are associated with this disease and these include late child-bearing, early menarche, smoking, history of mammary gland illness, postmenopausal obesity, and the Caucasian race [11]. The variation in presentation, outcomes, and etiologies of breast cancer highlights the importance of conducting further studies regarding its emanation and prevalence [11].

Lebanon is a small developing country in the Middle East, with a prominent breast cancer incidence [12,13]. Numerous regional and local conflicts have rendered the Ministry of Public Health (MOPH) in the country inactive and indolent [13,14,15,16,17,18,19,20,21]. Nevertheless, efforts were made in 2002 to restore action into the ministry’s National Cancer Registry (NCR), and as a result, an accurate count of all cancer cases present in Lebanon was established. The main purpose of the registry is to establish a cancer incidence reporting system and provide an archive of population-based cancer cases for investigators in the medical and public health sectors [22]. Throughout its data collection process, the NCR relies on two major systems—capture and recapture. The capture system is mainly reliant on the cases reported by physicians, either directly from their clinics or indirectly through the MOPH Drug Dispensing Center [22]. The recapture system relies on information from histopathologic and hematologic laboratories. The registry categorizes cases according to sex, age, and primary site of cancer. The NCR is reported to cover more than 90% of cancer cases in Lebanon, with the most recently published data encompassing the years 2005–2015 [23]. Cancer incidence data has been presented by the NCR on the official website of the MOPH, ever since [22,24,25].

Our study aimed to analyze the 11-year incidence rates of breast cancer in Lebanon from 2005, up to 2015. This study will also compare the incidence rates in Lebanon to the rates of other regional countries on the one hand, and selected countries from around the world on the other, and discuss the possible risk factors of this disease.

## 2. Materials and Methods

The Lebanese NCR was screened for the time period 2005–2015, to compute the age-specific incidence rates and the age-standardized rates (ASR(w)), expressed per 100,000 population. The age-specific incidence rate constituted the number of new cancer cases that occurred during a specific time-period, in a population of a specific age and sex group, divided by the number of midyear population of that age and sex group. The ASR(w) were the incidence rates that would have been observed in our studied population if they have had the same age composition as a reference population. Standardization is pivotal when comparing between different populations and age structures. In our study, we used Ferlay’s modified world population as the reference to compare the results of our population to those of different countries [26].

The computed age-specific rates and ASR(w) were analyzed using the joinpoint regression analysis with a significance level of 0.05. The joinpoint model provides information on the trends of breast cancer development, specifically the annual percent changes (APC) of breast cancer incidence, over the years studied. The computed age-specific rates and ASR(w) were then compared to those of the regional countries and other selected countries from different parts of the world. The selected countries were randomly chosen, using a random name picker, available on a website that allows the generation of country names, using the method of simple randomization [27]. These countries varied with respect to socioeconomic status and geographic location, with some being similar to Lebanon, and others being significantly different. As a result, this provided a global perspective as to where Lebanon stands with respect to breast cancer incidence. The data were obtained from two online databases—the Cancer Incidence in Five Continents (CI5XI) and (CI5Plus) [28,29]. These two databases emerged as a result of a collaboration between the International Agency for Research on Cancer (IARC) and the International Association of Cancer Registries (IACR).

With regards to the need for an Institutional Review Board (IRB) approval, our study is a descriptive epidemiological study with public data available online and published by the Lebanese Ministry of Public Health (https://www.moph.gov.lb/en). As such, it did not require an IRB approval.

## 3. Results

During the studied period (2005–2015), breast cancer was found to be the most prevalent cancer in Lebanon with a total of 22,357 cases reported, accounting for almost 37% of cancer cases among females, and 20% of all cancer cases. Lung cancer and colorectal cancer followed, with 10,459 and 9162 cases, respectively. An average of 2033 cases were reported every year. In general, the majority of patients were 35 years and older (90.3%).

**Figure 1:**
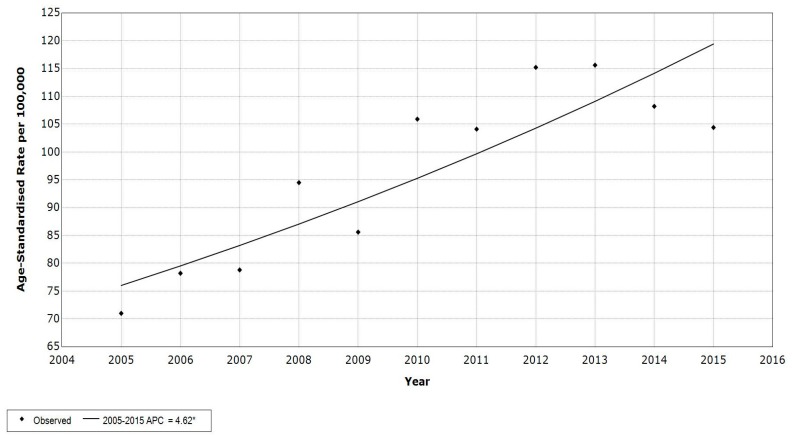
Age-Standardized Incidence rates (ASR(w)) (per 100,000) for breast cancer in females, in Lebanon 2005 to 2015.

**Figure 2:**
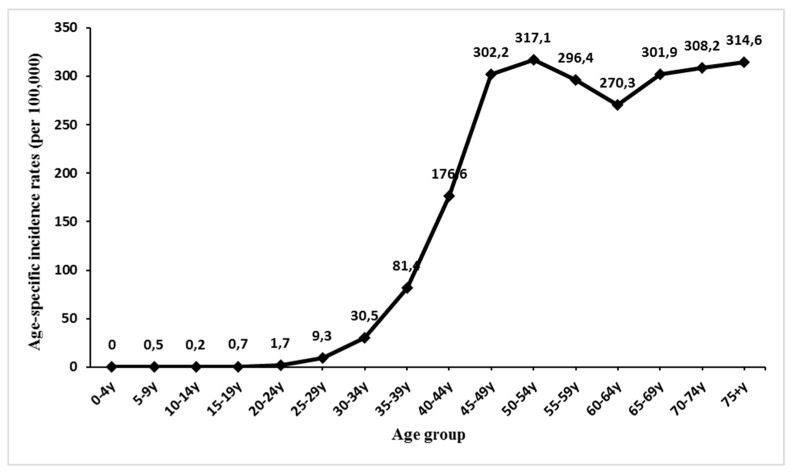
Average age-specific incidence rates (per 100,000 population) for breast cancer in Lebanese females, from 2005–2015.

The breast cancer ASR(w) among Lebanese females averaged 91.7 per 100,000 between 2005 and 2015. The rates ranged between 71 and 115.6 per 100,000. The annual percent change (APC) of breast cancer incidence rate, calculated using the joinpoint regression analysis, was found to be +4.6, indicating a significant increase along the years of our study (Figure 1). The APC of breast cancer age-specific rates significantly increased for the age groups 45–49 (*p* = 0.013, CI [1.5, 10.3]), 50–54 (*p* < 0.001, CI [4.2, 9.1]), 55–59 (*p* = 0.001, CI [2.5, 6.9]), 60–64 (*p* = 0.002, CI [1.9, 6.7]), 65–69 (*p* = 0.003, CI [2.2, 8.2]), 70–74 (*p* < 0.001, 95% CI [4.2, 7]), and 75+ years (*p* < 0.001, CI [3.4, 8.6]) (Table 1). The incidence rate of breast cancer increased with age (R^2^ = 0.85) and reached a peak of 317.1 per 100,000, for the 50–54 years age group (Figure 2).

Lebanon ranked first with respect to breast cancer ASR(w), followed by Malta and Kuwait with ASR(w)s of 79 per 100,000 and 56.1 per 100,000, respectively. In contrast, the Batna province of Algeria had the lowest ASR(w) of 25.9 per 100,000. When compared with breast cancer ASR(w)s of the randomly selected countries, Lebanon ranked among the highest, trailing only behind Denmark, which had an ASR(w) of 97.3 per 100,000; and surpassing both Germany and Italy which had ASR(w)s of 90.9 per 100,000 and 90.1 per 100,000 respectively (Table 2). The lowest ASR(w) of the selected countries belonged to Thailand at 24.8 per 100,000.

## 4. Discussion

Breast cancer was found to be the cancer with the greatest number of cases in the Lebanese population. ASR(w) among Lebanese females were found to be the highest in Middle-Eastern countries, and among the highest in the world [30].

Several social and health factors entail a variation in breast cancer incidence between different countries. The disparity in the implementation of screening procedures and awareness campaigns across different populations leads to a difference in reporting of breast cancer incidence [30,31,32]. Lesser degrees of awareness and rarer practice of screening procedures in regional countries like Egypt, Tunisia, and Saudi Arabia has led to the prevalence of advanced breast cancer cases in their population, but lower reported incidence rates [32,33,34]. On the other hand, Lebanon’s increased attention regarding awareness campaigns and screening procedures explain the higher incidence rates among its population [28]. In addition, the variation in cancer registration between different regions and countries is bound to cause a diversity in incidence rates. Some registries might include cancers in situ and of unknown behavior together with invasive cancers, and this yields different outcomes, when comparing countries to one another [30].

Many reproductive features present in Lebanese women predispose them to breast cancer more so than other countries [30]. The mean marital age for women in Lebanon has been on the rise for many years, reaching 28.3 in 2007. This is considerably high when compared to other countries like Egypt (22 in 2014), Iran (23.5 in 2011), Saudi Arabia (24.9 in 2007), Thailand (24.9 in 2010), Poland (26.6 in 2011) and Canada (26.9 in 2011) [35]. Moreover, fertility rates among Lebanese females have been decreasing steadily, when compared to other regional countries [36]. The fertility rate among Lebanese women was 1.7 births per woman in 2015, lower than Saudi Arabia (2.6 births per woman), Egypt (3.3 births per woman), and Jordan (3.4 births per woman) [35].

Smoking has been known to increase the risk of breast cancer as well, and it has been found to be high among the Lebanese population [30,37]. Its prevalence among Lebanese women was 27.2% in 2015, higher than Poland (23.8%), Canada (12%), Japan (11.2%), Saudi Arabia (1.8%), and Iran (0.8%). In addition, it has been shown that obesity is associated with higher breast cancer incidence among post-menopausal women [38]. A national population-based study once showed that prevalence of obesity and overweightness among Lebanese women is very high when compared to other countries, and this prevalence has been on the rise ever since [39]. Indeed, the prevalence of obesity among Lebanese females increased from 32.2% in 2005 to 36.5% in 2015; considerably higher than the prevalence of obesity among females in other countries like Iran (31.7%), Thailand (12.1%), Brazil (24.9%), and Japan (3.6%) [35]. The high prevalence of such health risk factors is bound to increase the incidence of breast cancer among Lebanese females.

Breast cancer incidence rates among Lebanese women increased significantly during the studied period. This might be attributed to the rise of the mean marital age, the drop in fertility rates, the high prevalence of obesity and smoking, the increased efficiency and improvement of NCR, and the rising use of mammography in the Lebanese population [30,35]. In addition, regional conflicts that occurred during the studied period caused a multitude of issues that affected cancer incidence in the country. The radioactive emissions caused by the use of depleted uranium warfare devices, along with the huge influx of refugees, have been reported to contribute to the rise of breast cancer incidence in Lebanon [40,41,42].

Many studies in Lebanon report that peak incidence of breast cancer occur at younger ages, as compared to other countries [43,44]. The MOPH in Lebanon recommends the start of breast cancer screening with mammography, at 40 years of age. In our study, breast cancer cases were nearly nil below the age group (25–29) and then started increasing as the age groups increased; more than 90% of the patients were 35 years and older. Supporting guidelines that sponsor earlier screening might help in earlier detection and might lessen the burden and severity of breast cancer cases in Lebanese women.

In addition, several studies in the literature explored the genetic susceptibility of the Lebanese population to breast cancer [45,46,47]. These studies reported a varied prevalence of *BRCA* mutations ranging between 5.6% to 12.5% of breast cancer cases, lower than those found in other populations. Given the genetic heterogeneity of this disease, this suggests the involvement of other genetic mutations in the pathogenesis of breast cancer in Lebanese women [48]. One study explored 45 Lebanese breast cancer patients and found nineteen mutations on thirteen different genes [45]. Another study explored the characteristics of breast cancer cases in Lebanon from 1990 to 2013 and reported that 8.3% of the cases were of the triple-negative subgroup [43]. Estrogen and progesterone receptors were found in 68.6% and 64.7% of the patients, respectively, and the Her2-neu was overexpressed in 30% [44]. Such information can be essential for the evaluation and classification of breast cancer cases in specific, and all cancers in general; and as such, additional studies that explore the genomic profile of Lebanese patients might prove vital in investigating the cause behind the surge of this disease [44,45,46,47,48,49,50].

It is worth mentioning that despite the high incidence of breast cancer among Lebanese women, breast cancer prognosis in the Lebanese population has been improving substantially. This is due to the improvement of the NCR, and the elevated drive towards earlier screening [51]. As a result, awareness towards cancer prevention and treatment increased, and the use of mammography has become more prevalent. In 2019, earlier stages of the disease constituted two-thirds of the cases in the Lebanese population, with survival rates exceeding 80%–90%. In addition, advanced and metastatic subtypes have decreased substantially [51]. Chemotherapy has been avoided in more than 50% of patients, and is increasingly being replaced by adjuvant hormonal therapy [51]. This serves to show that, in recent years, breast cancer prognosis and survival rates have improved in Lebanon [51].

## 5. Conclusions

Breast cancer rates among Lebanese women have been increasing for years and are amongst the highest in the region and worldwide. Many risk factors predispose Lebanese women to breast cancer and these include high mean marital age, low fertility rate, and the prevalence of obesity and smoking. Increased efficiency of the NCR in reporting breast cancer cases, higher awareness levels among the population, and the wider implementation of screening procedures contribute to the greater incidence of this disease as well. Future studies should aim at exploring the genomic profile of the Lebanese population, to propose better screening guidelines and prevention modalities.

## Figures and Tables

**Table 1:** Trend analysis for breast cancer age-specific rate (ASR) (per 100,000) in females by age group per year, Lebanon 2005–2015. *Annual Percent Change (APC) significantly different from zero.

Year	ASR (w)	0–4 y	5–9 y	10–14 y	15–19 y	20–24 y	25–29 y	30–34 y	35–39 y	40–44 y	45–49 y	50–54 y	55–59 y	60–64 y	65–69 y	70–74 y	75+ y
2005	71	0	0	0	1.6	0.5	9.7	31.6	75.3	149.8	175.9	246.3	202.8	207.2	215.5	226	217.5
2006	78.2	0	0	0.5	0.5	1.5	9	27.1	86.1	136.4	226.8	222.7	264.3	231.4	238.4	236.1	289.9
2007	78.8	0	0.6	0	0	1.6	4.2	35.8	80.9	171.2	213.2	246	271.1	227.6	209.9	258.1	225.5
2008	94.5	0	1	0.5	1	2.6	9.9	19.3	77.8	199.7	329.6	273.6	270.7	257.3	317.9	302.7	266.5
2009	85.6	0	0	0	0	1.5	10.8	29.5	87.7	167.9	280.2	237.1	239.2	240.2	290.9	261.5	283.8
2010	105.9	0	0	0	0	1.5	11.6	33.9	82	200.5	366.6	338.6	344.3	295.7	324.4	299.3	282.7
2011	104.1	0	0	0	0.5	0.5	6.8	26.4	91.6	184.8	362.8	349.6	314.1	244.5	313.4	340.9	374
2012	115.2	0	0	0	0	3.4	9.7	34.9	84.6	228.1	374.1	405	318.3	301.9	362	357.7	390.1
2013	115.6	0	0	0	0.4	0	7.1	29	87.6	177.2	388.7	399.8	346	361	393.2	359.5	341.8
2014	108.2	0.4	3.5	1.7	3.3	4.9	13.8	35	69	165.8	294.2	382.7	336.9	328.9	346.5	373.9	419.2
2015	104.4	0	0.3	0	0.4	0.7	10	32.8	73.2	160.8	311.7	387	352.2	278	308.7	374.9	369.8
APC	4.6 *	-	-	-	-	-	2.7	1.5	−0.5	1.5	5.8 *	6.6 *	4.7 *	4.3 *	5.1 *	5.6 *	6.0 *
*p*-value	<0.001	-	-	-	-	-	0.411	0.412	0.557	0.314	0.013	<0.001	0.001	0.002	0.003	<0.001	<0.001
CI	[2.7, 6.6]	-	-	-	-	-	[−4.2, 10.2]	[−2.4, 5.6]	[−2.4, 1.4]	[−1.6, 4.6]	[1.5, 10.3]	[4.2, 9.1]	[2.5, 6.9]	[1.9, 6.7]	[2.2, 8.2]	[4.2, 7]	[3.4, 8.6]

**Table 2:** Annual incidence rate (per 100,000) of breast cancer in females of different regional and selected countries * (excluding Nunavut, Quebec, and Yukon).

	Country	Years	ASR (w)	Age Groups															
				0–4 y	5–9 y	10–14 y	15–19 y	20–24 y	25–29 y	30–34 y	35–39 y	40–44 y	45–49 y	50–54 y	55–59 y	60–64 y	65–69 y	70–74 y	75+ y
**Regional Countries**	Algeria (setif)	2008–2011	44.7	-	-	0.4	0.6	2.9	9.5	34.9	72.7	115.7	134.4	124.6	103.7	134.7	122.3	76.4	57.9
	Algeria (Batna)	2008–2012	25.9	-	-	-	-	1.2	1.5	18.8	43.3	71.4	83.9	70.1	76.4	60.8	48.4	65.3	43.7
	Bahrain	2005–2012	53.6	-	-	-	-	0.5	8.1	25.6	51.5	82.6	125.9	177.1	171.2	200.5	194.6	159.1	150.2
	Egypt (Damietta)	2009–2012	53.4	0	0	0	0.375	1.6	7.35	31.3	50.65	90.15	115.5	140.83	194.9	207	221.05	123.55	158.975
	Iran (Golestan)	2008–2011	30.5	-	-	-	0.3	4.6	10.8	23.6	48.4	78	88.2	97.4	82.6	71	57.2	42.9	63.9
	Jordan	2008–2012	51	-	-	-	0.1	0.9	6.3	19.7	45.3	94.7	140.6	154	142	196.8	166.1	156.2	148.9
	Kuwait	2005–2012	56.1	-	-	-	-	1.0	7.7	21.2	37.2	77.9	144.5	145.0	208.7	218.7	223.3	191.9	185.8
	Lebanon	2005–2012	91.7	-	0.2	0.1	0.5	1.6	9	29.8	83.3	179.8	291.2	289.9	278.1	250.7	284.1	285.3	291.3
	Malta	2005–2012	79	-	1.2	-	0.9	0.9	4.2	21.8	48.2	111.0	183.4	197.8	277.2	294.8	303.6	327.7	410.2
	Qatar	2008–2012	53.8	-	-	-	-	1.9	10.4	21.4	46.1	115.4	106	180.9	217.8	131.6	177.9	230.8	127
	Saudi Arabia	2008–2012	24.5	-	-	-	-	1.4	6.8	14.6	23	48	64.9	55.9	69.1	89.5	99	78.5	58.8
**Selected Countries**	Turkey	2005–2012	43.8	-	-	0.1	0.1	1.0	7.9	22.8	48.1	86.9	115.0	122.5	125.4	142.6	148.8	136.3	128.1
	Cyprus	2005–2012	79.4	-	-	-	-	2.3	6.6	29.6	66.6	134.5	201.0	213.3	239.8	296.1	296.9	294.2	282.2
	Canada*	2005–2012	78.7	-	-	0.1	0.4	1.7	7.6	22.7	53.1	106.1	163.7	202.3	240.3	304.9	357.6	370.2	352.7
	Brazil, Goiania	2005–2012	53.8	0.3	-	-	0.2	2.8	6.1	25.2	43.7	83.5	121.3	139.3	170.4	173.9	256.5	198.7	205.9
	Thailand	2005–2012	24.8	-	-	-	0.2	0.7	4.9	13.7	32.2	50.9	76.2	80.4	79.5	70.7	62.3	58.7	46.6
	Denmark	2005–2012	97.3	-	-	-	-	0.8	8.1	28.9	55.6	109.7	181.2	279.2	328.7	430.8	491.4	363.5	383.6
	Germany	2005–2012	90.9	-	-	-	-	1.6	11.6	27.0	59.0	122.4	190.8	237.8	300.1	376.1	419.4	358.5	357.5
	Switzerland	2005–2012	86.9	-	-	-	0.2	0.7	7.6	26.6	60.4	114.3	195.1	239.7	258.2	332.2	409.1	374.1	352.3
	Japan	2005–2010	51.5	-	-	-	0.2	0.8	5.7	21.4	50.9	112.6	169.7	150.6	158.1	153.7	152.1	135.6	119.6
	Poland, Kielce	2005–2012	42.5	-	-	-	-	1.5	4.0	10.3	33.3	67.6	103.8	129.6	156.1	172.9	152.3	134.7	111
	Italy	2005–2010	90.1	-	-	-	0.1	1.8	8.7	28.3	69.5	149.8	235.2	257.5	269.1	322.8	369.0	307.3	330.1
	Costa Rica	2005–2011	41.3	-	-	-	0.2	1.3	4.0	13.6	30.4	58.3	86.7	105.4	137.3	154.2	177.3	178.9	179.0
